# Direct Vertebral Rotation (DVR) Does Not Improve Clinical and Radiological Results Compared to Differential Rod Contouring (DRC) in Patients Treated Surgically for Idiopathic Scoliosis

**DOI:** 10.3390/jcm12124091

**Published:** 2023-06-16

**Authors:** Wiktor Urbanski, Piotr Markowski, Rafal Zaluski, Anis Kokaveshi, Piotr Morasiewicz

**Affiliations:** 1 Department of Neurosurgery, Wroclaw Medical University, 50-367 Wroclaw, Poland; 2 Wojskowy Szpital Kliniczny, 50-981 Wroclaw, Poland; 3 Hygeia Hospital Tirana, 1051 Tirana, Albania; 4 Department of Orthopedics and Traumatology, Institute of Medical Sciences, University of Opole, 45-040 Opole, Poland

**Keywords:** adolescent idiopathic scoliosis, spinal surgery, apical rotation, direct vertebral rotation, differential rod contouring

## Abstract

Direct vertebral rotation (DVR) is the most widespread method to correct axial vertebral rotation. Differential rod contouring (DRC) also includes derotation, but not to the same extent as DVR. DVR requires additional surgical effort with potential consequences, which are absent in DRC; moreover, the data concerning the clinical benefits of apical derotation are not convincing. In the present study, clinical and radiological outcomes were compared in patients who underwent surgery for adolescent idiopathic scoliosis (AIS), having DVR and DRC vs. DRC only. In total, 73 AIS patients with curves of 40–85°, consecutively operated on by one surgeon, participated in this study and were followed up over 2 years. Scores from the SRS-22 questionnaire were analysed, the angles of trunk rotation (ATR) were measured with an inclinometer and a radiographic assessment of coronal and sagittal spinal profiles was conducted. In 38 cases, only DRC was performed, and in 35 DRC was performed and followed by DVR; the groups did not differ from an epidemiological point of view. Total SRS-22 scores after 2 years were similar in both groups (4.23 (±0.33) in DRC vs. 4.06 (±0.33) in DRC/DVR, *p* = 0.1). In all components of SRS-22, the differences were minor, with *p* being way above 0.05. The mean ATR in the DRC/DVR group was slightly smaller (8 ± 4°) than that of the DRC group (10 ± 5°), *p* = 0.16. Radiographic analysis did not show significant differences. The coronal curve was corrected by 66 ± 12% for DRC and 63 ± 15% for DVR, *p* = 0.28. Thoracic kyphosis in the DRC/DVR group increased by 1°, whereas in the DRC group the average kyphosis increased by 5° with a *p* value of 0.07. The complication rates were similar in both groups. This investigation did not show any advantages of the combination of DRC and DVR in scoliosis correction over DRC only, both radiologically and clinically, yet it affected intraoperative parameters, extending the operation time with only a minor increase in blood loss.

## 1. Introduction

With the development of modern implants and surgical techniques, more extensive and more efficient corrections of deformed spines are possible. Correcting all components of scoliosis—coronal, sagittal and axial deformity—is a well-established approach. The significance of coronal and sagittal corrections is known and documented in the literature [1]. However, less is known about the impact of axial correction. Derotation may be achieved in patients with scoliosis with apical vertebral rotation through several techniques. The most powerful technic developed to improve apical rotation in scoliosis surgery is direct vertebral rotation (DVR). There is research showing the radiographic improvement of vertebral rotations as well as enhanced coronal curve correction after DVR [2,3,4]. However, the whole procedure requires a longer operation time, it is associated with higher blood loss, and the associated manoeuvre puts significant direct stress on pedicle screws [5,6,7]. Furthermore, many authors have observed minor or no clinical benefits from derotation compared to non-derotation methods [8,9,10].

A less potent but gentler derotation treatment is differential rod contouring (DRC). An overbent convex rod and a flattened concave rod reduce apical vertebral rotation, which could be considered as indirect vertebral derotation [11,12].

Therefore, the objectives of this study were to compare clinical and radiological outcome after adolescent idiopathic scoliosis (AIS) surgery in patients having differential rod contouring (DRC) alone vs. patients with differential rod contouring (DRC) followed by direct vertebral rotation (DVR).

## 2. Material and Methods

**Figure 1:**
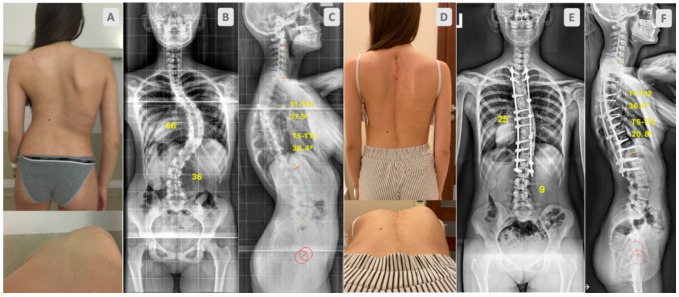
Sixteen-year-old girl with adolescent idiopathic scoliosis. Preoperative clinical pictures (**A**), standing radiographs, AP (**B**), lateral (**C**). Outcome at 2-year follow up after scoliosis correction surgery with DRC only. Clinical pictures (**D**), standing radiographs, AP (**E**), lateral (**F**).

**Figure 2:**
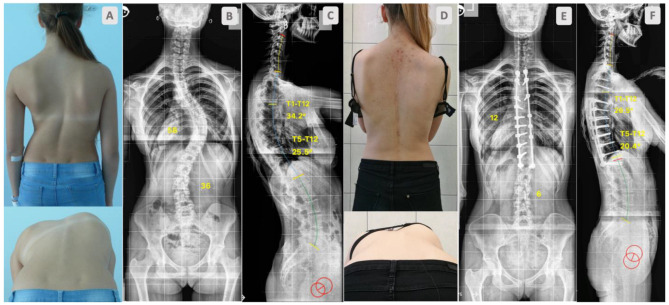
Fourteen-year-old girl with adolescent idiopathic scoliosis. Preoperative clinical pictures (**A**), standing radiographs, AP (**B**), lateral (**C**). Outcome at 2-year follow up after surgical scoliosis correction with DRC and DVR. Clinical pictures (**D**), standing radiographs, AP (**E**), lateral (**F**).

A total of 73 AIS patients, aged between 12 and 18 years old—Lenke 1 and 2 curves, with a curve magnitude 50–85°—were consecutively operated on by one surgeon. All completed at least a 2-year follow up. The participants were split into two groups: one comprising 38 patients who underwent correction with DRC only (Figure 1) and one comprised 35 patients for whom DRC correction was followed by the DVR manoeuvre (Figure 2).

Patients were followed up for at least 2 years and had full clinical and radiographic assessments. The clinical evaluation assessed health-related quality of life with the SRS-22r questionnaire and measuring the angles of trunk rotation (ATR) with an inclinometer at the apex of scoliosis. The measures were obtained with patients standing upright, their feet parallel and leaning forward while keeping their legs straight. The inclinometer was then centred over the spinous process at the apex of scoliosis, moving cranially and caudally, with the highest measurement in the thoracic region being recorded. To ensure the best reliability and repeatability, one author only (PM) performed the examinations. Whole spine standing radiographs—obtained preoperatively, immediately after surgery and after two years—were analysed. Coronal curves were measured using the Cobb method, and the extent (percentage) of correction following surgery and kyphosis was measured between T1 and T12 endplates. Curve flexibility was calculated on prone bending films.

The levels of instrumentation in general were determined according to the guidelines published by Lenke [13]. Both groups had similar patterns of screw distribution; caudal and cranial ends were more densely supplied with screws, as was the apex of scoliosis (3 levels at least). At concavity, CoCr rods were used, on convexity titanium alloy (Ti6Al4V).

**Figure 3:**
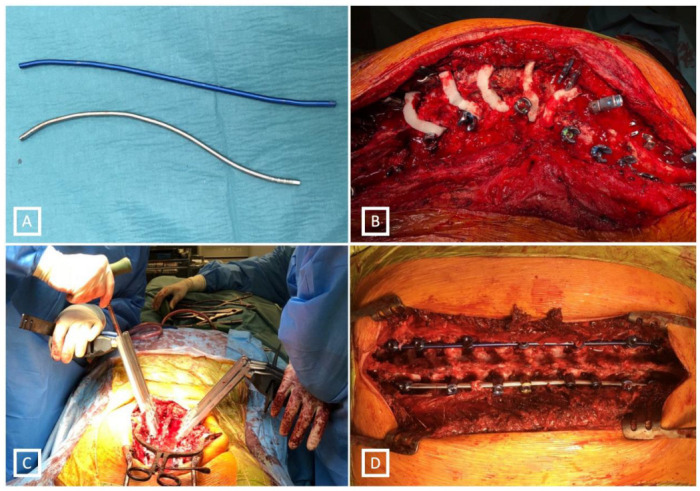
Techniques of deformity correction. (**A**) Differential rod contouring (DRC), the blue (**upper**) flattened, silver (**lower**) rod with exaggerated contouring. (**B**) Ponte osteotomies in 5 levels. (**C**) En bloc direct vertebral rotation (DVR). (**D**) Both rods applied with final correction.

When performing the differential rod contouring (DRC) method, the concave rod was over-contoured and the convex one under-contoured. Only partial facetectomies were performed: an inferior articular process was removed at the superior vertebra and no other osteotomies were conducted in any case of DRC (Figure 3).

In patients who had DRC with subsequent *en bloc* direct vertebral rotation (DVR), first posterior column osteotomies PCOs (Ponte type osteotomies) were performed [3,4]. PCO consisted of the complete resection of both facets, ligamentum flavum. All operations were conducted by the same surgeon (WU) (Figure 3).

The same protocol of tranexamic acid was applied in each case in both groups (bolus 15 mg/kg prior to surgery with continuous 2 mg/kg/h to skin closure), and the mean arterial blood pressure was maintained at approximately 70–80 mm Hg. Only absorbable haemostatic gelatin sponges were used in case of PCO, but no other local haemostatic agents were. No intraoperative cell salvage system was used in any case. The length of the operations was noted as well as estimated blood loss (EBL) during all surgeries.

Data are presented as the mean with standard deviation (±). Data and comparisons between groups were statistically analysed using t-student and Mann–Whitney tests. U—the value of Mann–Whitney test—was used when both groups had fewer than 20 measurements each, while Z—the value of Mann–Whitney test—was used when one of the groups had ≥20 measurements. In the case of the non-compliance homogeneity of variance and/or normal distribution (*p* > 0.05), a t Student test could not be used and thus the Mann–Whitney test was selected. A *p* value of less than 0.05 was statistically significant. The institutional review board of the institution approved the research.

## 3. Results

The age, sex, curve magnitude, curve flexibility, screw density, and surgery extension of patients were similar in the analysed groups, as presented in Table 1. The results of the SRS-22r questionnaires display similar scores in both groups. However, differences in satisfaction from management were the most distinct, with higher numbers in DRC only than DRC and DVR, as presented in Table 2.

The radiographic analysis showed a similar amount of coronal correction in both groups, with mean corrections of scoliosis of 66% (±12) for DRC and of 63% (±15) for DRC and DVR, with a *p* value of 0.28 (Table 3).

Mean T1–T12 kyphosis was higher preoperatively in DRC and DVR 37° (±10) than in DRC 32° (±11), *p* = 0.042; however, the post-surgery kyphosis magnitudes were similar in both groups—DRC 37° (±8) vs. DRC/DVR 39° (±9), *p* = 0.424. In both groups, mean kyphosis increased, although the mean growth was greater in the DRC group (by 5°) than in the DRC/DVR group (by 1°), with a *p* value of 0.07 (Table 3).

**Figure 4:**
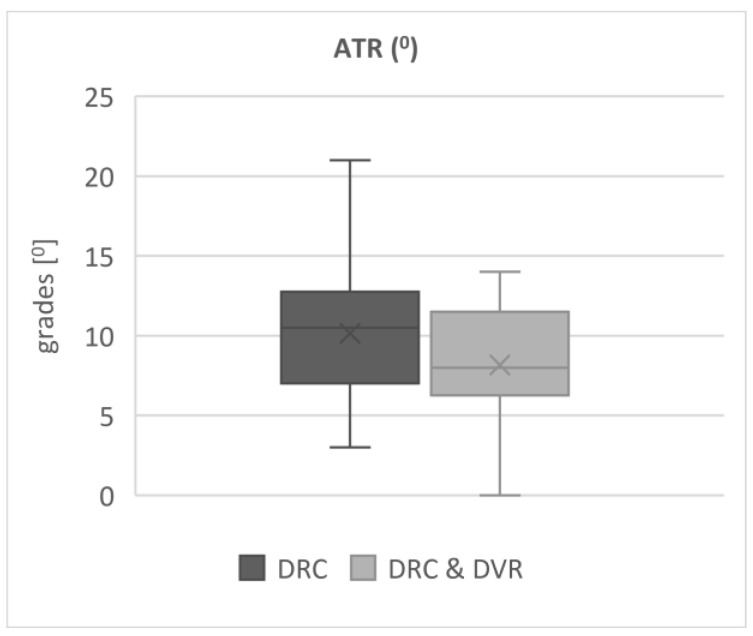
The average angle of trunk rotation after 2-year follow up in both groups.

The average angle of trunk rotation was only slightly higher in the DRC group 10° (±5) than the DRC and DVR 8° (±4) group, with a *p* value of 0.16 (Figure 4).

**Figure 5:**
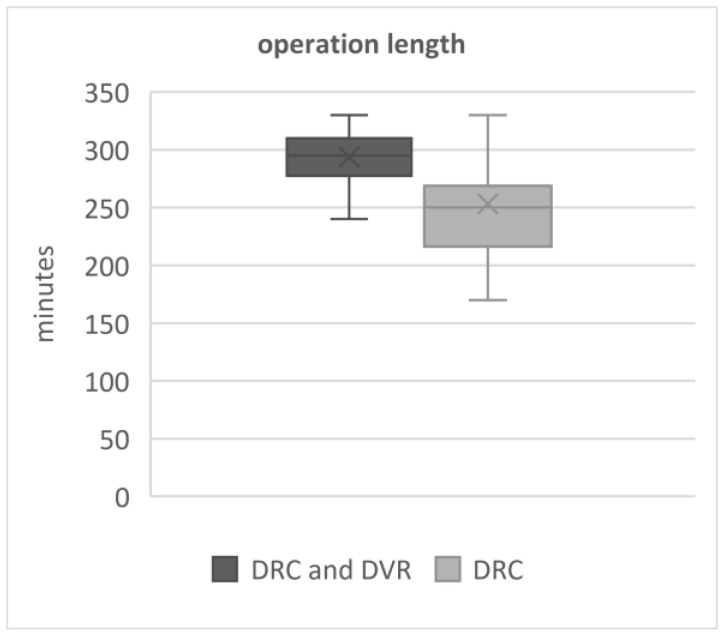
Length of the operations in both groups.

We analysed the surgery time and blood loss and noticed that the mean operation time was longer in the DRC/DVR group—293 min (±36)—than in the DRC group—253 min (*p* value 0.0001), Figure 5.

**Figure 6:**
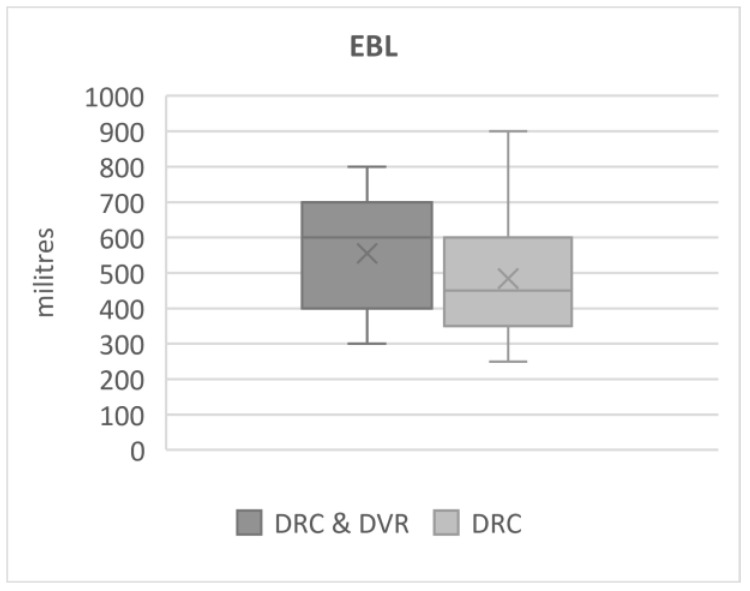
Estimated blood loss.

Estimated blood loss (EBL) was higher in the DRC and DVR group (555 mL) (±151.5) than DRC alone (489 mL) (±163.7), but the differences were statistically insignificant, *p* = 0.06 (Figure 6).

## 4. Discussion

DVR was introduced to compliment 3D correction, improve rib hump reduction and minimise fusion extension; however, the benefits from the procedure have not been determined so far. According to some authors, DVR may be related to hypokyphosis in the thoracic spine, increased risk of screw pull-out, longer operative times and greater blood loss [6,7,9,14,15]. The presented outcomes of the surgical treatment of idiopathic scoliosis have not revealed any significant differences between patients corrected only by DRC vs. patients having DRC followed by DVR. In the follow up, patients’ reported-outcome scores were high and did not differ between groups. Radiographic analysis showed substantial but similar corrections in the coronal as well as sagittal planes in both groups. Yet, when DVRs with PCOs were performed, the operations took more time, with slightly greater blood loss. 

Good clinical results, including patients’ own assessments, are the most important goal of AIS surgical treatment; satisfaction from the treatment over a long follow up is more important than minor differences in Cobb measurements or persisting apical rotation. Most of the articles on derotation techniques focus on radiological data, which do not necessarily have a direct association with patients’ satisfaction. There are only few papers comparing patient-reported outcomes with apical derotation and without. The available literature shows no evidence of the benefits from derotation; most of the papers show comparable scores for patients being or not being subjected to derotation manoeuvres [8,16,17].

We investigated patients’ quality of life and satisfaction in both approaches; we did not notice any differences that could suggest an advantage of one method above the other. Moreover, the differences in particular sections of SRS-22 (Table 2) did not satisfy the criteria of Minimum Clinically Important Difference (MCID), published by Carreon et al. [18].

Axial vertebral correction and its contribution to rib hump reduction are still debatable. According to many authors, rib hump deformity most likely results from asymmetric rib growth rather than from vertebral rotation, and ATR measurements correlate with apical axial vertebral rotation but only up to a certain point [19,20]. The residual rib hump exists even if significant vertebral derotation is carried out, due to the fixed deformity of the ribs and also the fact that ribs may continue to grow asymmetrically postoperatively if the patient still has growth potential, a phenomenon resulting in the relapse of the thoracic deformity [19,20,21]. In clinical papers, authors have not found any beneficial effects of DVR on rib hump reduction compared to methods that include no derotation [16,22]. Additionally, obtaining reliable and repeatable ATR measurements may be difficult; therefore, the authors considered these data rather as an additional outcome measure. The application of 3D measurement techniques (e.g., EOS system with 3D reconstructions of the ribcage) might prove more valid in an assessment of rib hump correction. Moreover, Seki et al. demonstrated that DRC also decreases the rib hump [23]; however, except for the data presented in this paper, no comparison of DVR vs. DRC has been performed so far. In our study, both methods provided satisfactory rib hump reduction, although the rib hump was smaller in DVR/DRC patients than in DRC by 2°, but this difference proved to be statistically insignificant (*p* = 0.16) and clinically undetectable (Figure 4). 

According to many authors, DVR provides improved coronal correction [4,24]; however, it may also have a lordotic effect on the thoracic spine [15]. From previous papers, it is known that the shift towards pedicle screws from hooks, especially to high-density screw constructs, contributed to the decrease in kyphosis and improvement of the coronal and axial plane. According to Acaroglu et al., due to shape of the discs and vertebral bodies in AIS, posterior surgery cannot correct all three planes at the same time [25]. The anterior column at the apex is longer than the posterior column in AIS, and this discrepancy mostly concerns the discs, less so the vertebral bodies [26]. The additional anterior length, rotated to the midline, creates a thoracic hypokyphosis/lordosis at the apex. What has recently also been confirmed by Hershkovitz et al. is that a significant correction in the coronal plane is associated with postoperative hypokyphosis [26,27]. Therefore, fewer significant coronal or axial corrections may be required to achieve better kyphosis restoration. Substantial axial correction is not necessary in terms of hump reduction; as mentioned earlier, the residual rib hump exists even if significant vertebral derotation is carried out. The data presented in this study showed similar kyphosis magnitudes in both groups at follow up (DRC 37° ± 8 vs. DRC/DVR 39° ± 9, *p* = 0.424). However, preoperative kyphosis was smaller in the DRC/DVR group; hence, the mean growth was greater in the DRC group (by 5°) than in the DRC/DVR group (by 1°), with a *p* value of 0.07 (Table 3).

No advantage in terms of the amount of coronal correction achieved was noticed between groups (Table 3). We presented similar groups in terms of curve magnitude (58° vs. 62° *p* = 0.098) and flexibility (36% vs. 33% *p* = 0.372), and besides the additional release of the spinal column and DVR, the group has not shown better results (Figure 1 and Figure 2). This is in opposition to earlier papers [4,22]. Although, in this study, CoCr rods were used; therefore, it seems that using CoCr 5.5 rods in cases of moderate curves may have a great effect on coronal correction over PCOs and DVRs [28]. However, the literature is not consistent on this topic; many authors observed no differences in outcomes between titanium or CoCr rods [29]; some noticed some in the sagittal plane [30].

The question is also opened as to whether DVR may reduce the extent of spinal fusion. The early papers from Lee and Suk et al. [2,31] suggest that apical derotation may reduce the extent of fusion; however, no strong evidence has emerged so far. On the other hand, the derotation of the last instrumented vertebra (LIV) may affect the length of fusion. A reduction of at least 50% of the rotation in LIV may ensure a good and durable long-term effect for lumbar uninstrumented spine [32,33]. In the present study, we did not assess lumbar curves nor the extent of fusion in both groups, although no clinically relevant addition was noted in any patient.

The DVR manoeuvre itself does not take a substantial amount of surgical time or cause significant additional bleeding. Yet, the additional time and bleeding necessary for PCOs and DVR makes a difference [34,35,36]. According to the presented data, the EBL was slightly higher in DVR/DRC with PCOs than DRC by an average of nearly 66 mL (*p* = 0.06), though surgeries in DVR/DRC with PCOs consumed much more time, on average by 40 min (*p* = 0.0001). Nearly 70 mL of blood loss may not have any clinical implications; a 40 min operation on the other hand can make a difference (Figure 5 and Figure 6).

Several authors pointed out the higher complication rate in patients having DVR and PCOs [37]; yet, considering DVR alone, the complication rate is not significantly elevated compared to non-DVR patients [24]. It has been shown that substantial forces applied on the screws during DVR may loosen them or even fracture the pedicle, causing screw pull-out and implant migration [6]; nevertheless, the clinical relevance of this has not been proven. Adding PSOs to DVR changes circumstances; as Floccari et al. demonstrated, patients having Ponte osteotomies developed over five times more complications [37]. Therefore, another controversy emerged; performing Ponte osteotomies improves the effectiveness of DVR [38,39], but at the same time raises complication rates.

This study has several limitations. This a retrospective study with only a small number groups. After a period of routine performance of PCOs, correction with DRC and then DVR—this consecutive series of patients formed the DRC/DVR group—one author [WU] abandoned the procedure in favour of DRC only without PCOs, which has become the standard approach in moderate AIS cases. The topic certainly needs more studies on larger groups, and prospective long-term observations with properly randomised groups.

In summary, the data presented showed comparable good results of surgically treated patients with moderate cases of adolescent idiopathic scoliosis over more than two years of follow up. The analysis of clinical and radiographic outcomes did not show any advantages of correction with DRC and DVR proceeded by posterior column osteotomies (PCOs) over indirect vertebral rotation or differential rod contouring (DRC) without osteotomies. Nevertheless, DRC/DVR compared to DRC only affected intraoperative parameters; it caused slightly higher blood loss, but significantly extended the operation time.

## Figures and Tables

**Table 1:** Epidemiology.

	DRC (*n* = 38)	DRC and DVR (*n* = 35)	*p* Value
**Age (years)**	15.6 (±1.5)	15.5 (±1.6)	0.814
**Sex (males/females)**	3/38	3/35	0.917
**Main curve magnitude**	62 (±10)	58 (±14)	0.098
**Coronal curve flexibility (%)**	33 (±14)	36 (±12)	0.372
**Number of instrumented levels**	11.8 (±1.7)	11.1 (±2)	0.107
**Screw density**	78 (±7.2)	77.6 (±6.3)	0.774

**Table 2:** Results of SRS 22r questionnaire after 2-year follow up.

SRS-22r	Total	Function	Pain	Self-Image	Mental Health	Satisfaction from Management
**DRC**	4.23 (±0.33)	4.26 (±0.48)	4.36 (±0.48)	4.14 (±0.54)	3.88 (±0.63)	4.86 (±0.27)
**DRC and DVR**	4.06 (±0.33)	4.17 (±0.59)	4.13 (±0.55)	4.03 (±0.59)	3.68 (±0.51)	4.68 (±0.35)
***p* value**	0.101	0.579	0.137	0.509	0.263	0.065

**Table 3:** Mean percentage of coronal curve correction at 2-year follow up. Thoracic kyphosis, prior to operation and at 2-year follow up.

		DRC	DRC/DVR	*p*
Coronal curve	Pre op [°]	62 (±10)	58 (±14)	0.098
	Post op [°]	21 (±8)	22 (±11)	0.601
	% of correction	66 (±12)	63 (±15)	0.28
Kyphosis T1–T12	Pre op [°]	32 (±11)	37 (±10)	0.043
	Post op	37 (±8)	39 (±9)	0.424

## Data Availability

The data presented in this study are available on request from the corresponding author.

## References

[1] Tambe A.D., Panikkar S.J., Millner P.A., Tsirikos A.I. (2018). Current concepts in the surgical management of adolescent idiopathic scoliosis. Bone Jt. J..

[2] Lee S.M., Suk S.I., Chung E.R. (2004). Direct vertebral rotation: A new technique of three-dimensional deformity correction with segmental pedicle screw fixation in adolescent idiopathic scoliosis. Spine.

[3] Chang M.S., Lenke G.L. (2009). Vertebral derotation in adolescent idiopathic scoliosis. Oper Tech. Orthop..

[4] Urbanski W., Wolanczyk M.J., Jurasz W., Kulej M., Morasiewicz P., Dragan S.L., Sasiadek M., Dragan S.F. (2017). The impact of direct vertebral rotation (DVR) on radiographic outcome in surgical correction of idiopathic scoliosis. Arch. Orthop. Trauma Surg..

[5] Sariyilmaz K., Ozkunt O., Gemalmaz H.C., Cingoz T., Pehlivanoglu T., Aksoy T., Kaya O., Baydogan M., Dikici F. (2020). Direct vertebral rotation significantly decreases the pullout strength of the pedicle screw: A biomechanical study in adult cadavers. J. Pediatr. Orthop. B.

[6] Badve S.A., Ordway N.R., Albanese S.A., Lavelle W.F. (2015). Toward a better understanding of direct vertebral rotation for AIS surgery: Development of a multisegmental biomechanical model and factors affecting correction. Spine J..

[7] Hicks J., Singla A., Arlet V. (2009). 145. Complications of Pedicle Screw Fixation in Scoliosis Surgery: A Systematic Review. Spine J..

[8] Di Silvestre M., Lolli F., Bakaloudis G., Maredi E., Vommaro F., Pastorelli F. (2012). Apical vertebral derotation in the posterior treatment of adolescent idiopathic scoliosis: Myth or reality?. Eur. Spine J..

[9] Rushton P.R.P., Grevitt M.P. (2014). Do vertebral derotation techniques offer better outcomes compared to traditional methods in the surgical treatment of adolescent idiopathic scoliosis?. Eur. Spine J..

[10] Kim G.-U., Yang J.H., Chang D.-G., Suk S.-I., Suh S.-W., Song K.-S., Nam K.-Y., Oh I.-S., Park H.-Y., Kim S.-I. (2019). Effect of Direct Vertebral Rotation in Single Thoracic Adolescent Idiopathic Scoliosis: Better 3-Dimensional Deformity Correction. World Neurosurg..

[11] Seki S., Kawaguchi Y., Nakano M., Makino H., Mine H., Kimura T. (2015). Rod rotation and differential rod contouring followed by direct vertebral rotation for treatment of adolescent idiopathic scoliosis: Effect on thoracic and thoracolumbar or lumbar curves assessed with intraoperative computed tomography. Spine J..

[12] Wang X., Boyer L., Le Naveaux F., Schwend R.M., Aubin C.-E. (2016). How does differential rod contouring contribute to 3-dimensional correction and affect the bone-screw forces in adolescent idiopathic scoliosis instrumentation?. Clin. Biomech..

[13] Lenke L.G. (2007). The Lenke Classification System of Operative Adolescent Idiopathic Scoliosis. Neurosurg. Clin. N. Am..

[14] Mladenov K.V., Vaeterlein C., Stuecker R. (2011). Selective posterior thoracic fusion by means of direct vertebral derotation in adolescent idiopathic scoliosis: Effects on the sagittal alignment. Eur. Spine J..

[15] Watanabe K., Nakamura T., Iwanami A., Hosogane N., Tsuji T., Ishii K., Nakamura M., Toyama Y., Chiba K., Matsumoto M. (2012). Vertebral derotation in adolescent idiopathic scoliosis causes hypokyphosis of the thoracic spine. BMC Musculoskelet. Disord..

[16] Tang X., Zhao J., Zhang Y. (2014). Radiographic, clinical, and patients’ assessment of segmental direct vertebral body derotation versus simple rod derotation in main thoracic adolescent idiopathic scoliosis: A prospective, comparative cohort study. Eur. Spine J..

[17] Sun L., Song Y., Liu L., An Y., Zhou C., Zhou Z. (2013). Bilateral apical vertebral derotation technique by vertebral column manipulation compared with vertebral coplanar alignment technique in the correction of lenke type 1 idiopathic scoliosis. BMC Musculoskelet. Disord..

[18] Carreon L.Y., Kelly M.P., Crawford C.H., Baldus C.R., Glassman S.D., Shaffrey C.I., Bridwell K.H. (2017). SRS-22R Minimum Clinically Important Difference and Substantial Clinical Benefit after Adult Lumbar Scoliosis Surgery. Spine Deform..

[19] Grivas T.B., Vasiliadis E.S., Mihas C., Savvidou O. (2007). The effect of growth on the correlation between the spinal and rib cage deformity: Implications on idiopathic scoliosis pathogenesis. Scoliosis.

[20] Grivas T.B., Vynichakis G., Chandrinos M., Mazioti C., Papagianni D., Mamzeri A., Mihas C. (2021). Morphology, Development and Deformation of the Spine in Mild and Moderate Scoliosis: Are Changes in the Spine Primary or Secondary?. J. Clin. Med..

[21] Jankowski P.P., Yaszay B., Cidambi K.R., Bartley C.E., Bastrom T.P., Newton P.O. (2018). The Relationship between Apical Vertebral Rotation and Truncal Rotation in Adolescent Idiopathic Scoliosis Using 3D Reconstructions. Spine Deform..

[22] Mattila M., Jalanko T., Helenius I. (2013). En Bloc Vertebral Column Derotation Provides Spinal Derotation but no Additional Effect on Thoracic Rib Hump Correction as Compared with no Derotation in Adolescents Undergoing Surgery for Idiopathic Scoliosis with Total Pedicle Screw Instrumentation. Spine.

[23] Seki S., Newton P.O., Yahara Y., Makino H., Nakano M., Hirano N., Kawaguchi Y., Kimura T. (2018). Differential rod contouring is essential for improving vertebral rotation in patients with adolescent idiopathic scoliosis: Thoracic curves assessed with intraoperative CT. Spine.

[24] Son S.M., Choi S.H., Goh T.S., Park W., Lee J.S. (2019). Efficacy and Safety of Direct Vertebral Rotation in the Surgical Correction of Scoliosis: A Meta-Analysis. World Neurosurg..

[25] Acaroglu E., Doany M., Cetin E., Castelein R. (2019). Correction of rotational deformity and restoration of thoracic kyphosis are inversely related in posterior surgery for adolescent idiopathic scoliosis. Med. Hypotheses.

[26] Schlösser T.P., van Stralen M., Brink R.C., Chu W.C.W., Lam T.-P., Vincken K.L., Castelein R.M., Cheng J.C.Y. (2014). Three-Dimensional Characterization of Torsion and Asymmetry of the Intervertebral Discs versus Vertebral Bodies in Adolescent Idiopathic Scoliosis. Spine.

[27] Hershkovich O., D’souza A., Rushton P.R.P., Onosi I.S., Yoon W.W., Grevitt M.P. (2020). Essential lordosis revisited. Bone Jt. J..

[28] Ruffilli A., Fiore M., Viroli G., Barile F., Manzetti M., Martikos K., Greggi T., Faldini C. (2022). 5.5-mm Cobalt-Chrome vs 6-mm Titanium Alloy Rods in Surgical Treatment of Lenke 1 Adolescent Idiopathic Scoliosis with High-Density Pedicle Screws and Direct Vertebral Rotation on Differently Shaped Rods: A Retrospective Comparative Cohort Study. Int. J. Spine Surg..

[29] Sabah Y., Clément J.-L., Solla F., Rosello O., Rampal V. (2018). Cobalt-chrome and titanium alloy rods provide similar coronal and sagittal correction in adolescent idiopathic scoliosis. Orthop. Traumatol. Surg. Res..

[30] Ohrt-Nissen S., Dahl B., Gehrchen M. (2018). Choice of Rods in Surgical Treatment of Adolescent Idiopathic Scoliosis: What Are the Clinical Implications of Biomechanical Properties?—A Review of the Literature. Neurospine.

[31] Suk S.I., Lee S.M., Chung E.R., Kim J.-H., Kim S.-S. (2005). Selective thoracic fusion with segmental pedicle screw fixation in the treatment of thoracic idiopathic scoliosis: More than 5-year follow-up. Spine.

[32] Pasha S., Cahill P.J., Flynn J.M., Sponseller P., Newton P.O., Harms Study Group (2018). Relationships between the Axial Derotation of the Lower Instrumented Vertebra and Uninstrumented Lumbar Curve Correction: Radiographic Outcome in Lenke 1 Adolescent Idiopathic Scoliosis with a Minimum 2-Year Follow-up. J. Pediatr. Orthop..

[33] Chang D.-G., Suk S.-I., Kim J.-H., Song K.-S., Suh S.-W., Kim S.-Y., Kim G.-U., Yang J.H., Lee J.-H. (2020). Long-term Outcome of Selective Thoracic Fusion Using Rod Derotation and Direct Vertebral Rotation in the Treatment of Thoracic Adolescent Idiopathic Scoliosis: More than 10-Year Follow-up Data. Clin. Spine Surg..

[34] Huang Z., Wang Q., Yang J., Yang J., Li F. (2016). Vertebral Derotation by Vertebral Column Manipulator Improves Postoperative Radiographs Outcomes of Lenke 5C Patients for Follow-up of Minimum 2 Years. Clin. Spine Surg. A Spine Publ..

[35] Samdani A.F., Bennett J.T., Singla A.R., Marks M.C., Pahys J.M., Lonner B.S., Miyanji F., Shah S.A., Shufflebarger H.L., Newton P.O. (2015). Do Ponte Osteotomies Enhance Correction in Adolescent Idiopathic Scoliosis? An Analysis of 191 Lenke 1A and 1B Curves. Spine Deform..

[36] Halanski M.A., Cassidy J.A. (2013). Do Multilevel Ponte Osteotomies in Thoracic Idiopathic Scoliosis Surgery Improve Curve Correction and Restore Thoracic Kyphosis?. J. Spinal Disord. Tech..

[37] Floccari L.V., Poppino K., Greenhill D.A., Sucato D.J. (2021). Ponte osteotomies in a matched series of large AIS curves increase surgical risk without improving outcomes. Spine Deform..

[38] Sangiorgio S.N., Borkowski S.L., Day M.J., Ho N.C., Knutsen A., Scaduto A.A., Bowen R.E., Ebramzadeh E. (2020). Increasing loads and diminishing returns: A biomechanical study of direct vertebral rotation. Spine Deform..

[39] Seki S., Yahara Y., Makino H., Kawaguchi Y., Kimura T. (2018). Selection of posterior spinal osteotomies for more effective periapical segmental vertebral derotation in adolescent idiopathic scoliosis–An in vivo comparative analysis between Ponte osteotomy and inferior facetectomy alone. J. Orthop. Sci..

